# Total Beta-Adrenoceptor Knockout Slows Conduction and Reduces Inducible Arrhythmias in the Mouse Heart

**DOI:** 10.1371/journal.pone.0049203

**Published:** 2012-11-01

**Authors:** Florian Stöckigt, Klara Brixius, Lars Lickfett, René Andrié, Markus Linhart, Georg Nickenig, Jan Wilko Schrickel

**Affiliations:** 1 Department of Medicine-Cardiology, University Hospital of Bonn, Bonn, Germany; 2 German Sportuniversity Cologne, Cologne, Germany; University Hospital of Würzburg, Germany

## Abstract

**Introduction:**

Beta-adrenoceptors (β-AR) play an important role in the neurohumoral regulation of cardiac function. Three β-AR subtypes (β_1_, β_2_, β_3_) have been described so far. Total deficiency of these adrenoceptors (TKO) results in cardiac hypotrophy and negative inotropy. TKO represents a unique mouse model mimicking total unselective medical β-blocker therapy in men. Electrophysiological characteristics of TKO have not yet been investigated in an animal model.

**Methods:**

In vivo electrophysiological studies using right heart catheterisation were performed in 10 TKO mice and 10 129SV wild type control mice (WT) at the age of 15 weeks. Standard surface ECG, intracardiac and electrophysiological parameters, and arrhythmia inducibility were analyzed.

**Results:**

The surface ECG of TKO mice revealed a reduced heart rate (359.2±20.9 bpm vs. 461.1±33.3 bpm; p<0.001), prolonged P wave (17.5±3.0 ms vs. 15.1±1.2 ms; p = 0.019) and PQ time (40.8±2.4 ms vs. 37.3±3.0 ms; p = 0.013) compared to WT. Intracardiac ECG showed a significantly prolonged infra-Hisian conductance (HV-interval: 12.9±1.4 ms vs. 6.8±1.0 ms; p<0.001). Functional testing showed prolonged atrial and ventricular refractory periods in TKO (40.5±15.5 ms vs. 21.3±5.8 ms; p = 0.004; and 41.0±9.7 ms vs. 28.3±6.6 ms; p = 0.004, respectively). In TKO both the probability of induction of atrial fibrillation (12% vs. 24%; p<0.001) and of ventricular tachycardias (0% vs. 26%; p<0.001) were significantly reduced.

**Conclusion:**

TKO results in significant prolongations of cardiac conduction times and refractory periods. This was accompanied by a highly significant reduction of atrial and ventricular arrhythmias. Our finding confirms the importance of β-AR in arrhythmogenesis and the potential role of unspecific beta-receptor-blockade as therapeutic target.

## Introduction

The myocardial beta-adrenergic receptors (β-AR) play a pivotal role in regulating cardiac autonomic function. Three β-AR subtypes have been described and identified by molecular cloning so far: β_1_ [1], β_2_ [2] and β_3_ [3]. Both the β_1_- and β_2_-receptor are Gs-protein-coupled and mediate their actions by the adenylylcyclase-protein kinase A cascade with cyclic AMP as a second messenger. The downstream mechanism includes phosphorylation of phospholamban and sarcoplasmatic/endoplasmatic reticulum calcium ATPase (SERCA) that enhances intracellular calcium dynamics [4]. Stimulation by catecholamines predominantly leads to positive inotropic, chronotropic and lusitropic responses in the heart [5]. The stimulation of the β_2_-AR is additionally associated with the regulation of cardiac growth and remodelling [6]: a cardioinhibitory pathway via the Gi-protein [7] as well as a mutual interference with pathways regulating gene transcription have recently been described [8]. β_3_-adrenergic stimulation is connected to nitric oxide liberation by means of endothelial nitric-oxide-synthase (eNOS) activation [9] as well as inducible (iNOS) and neuronal nitric-oxide-synthase (nNOS) and has a negative inotropic effect at high levels of sympathetic stimulation [10].

Chronic β-adrenergic stimulation, e.g. in patients with dilated cardiomyopathy and heart failure, results in a down-regulation of the β-AR [11] and may lead to electrophysiological disturbances of the myocardium, entailing tachyarrhythmia and sudden cardiac death [12]. Beta-blocking agents are used in these patients and other cardiovascular diseases such as coronary heart disease, in which some clinically used unselective beta-blockers not only affect β_1_- and β_2_-AR but also are associated with effects related to the β_3_-AR [13]. Nebivolol is one example that possesses a β_1_-blocking power with additional β_3_-agonistic effects [14]. Selective β_1_-blockers are commonly given with higher priority than non-selective beta-blockers since several side effects (e.g. bronchospasm) [15] have been ascribed to the blockade of β_2_-AR and β_1_-blockers have been proven effective to treat heart failure in large clinical trials [16].

**Figure 1:**
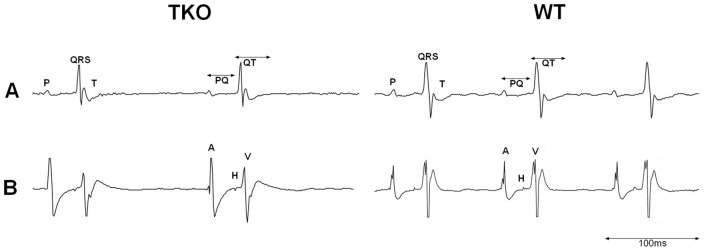
Representative ECG recordings of TKO and WT mice. A: Surface ECG. B: Intracardiac ECG at His–bundle level (A: first intracardiac atrial signal. H: His signal. V: first intracardiac ventricular signal).

Jiminez et al. [17] generated a viable mouse model with a total β_1/2/3_-AR knockout (TKO  =  β_1/2/3_-KO  =  β_1_^−/−^β_2_^−/−^β_3_^−/−^). These mice show cardiac hypotrophy and negative inotropy accompanied by a decreased SERCA 2a activity [18]. This mouse model represents an excellent opportunity to further study the electrophysiological characteristics of β-AR. Beyond that, this knockout model mimics a beta-blocker pharmacotherapy affecting not only the positive inotropic effects of β_1_- and β_2_-AR but also the β_3_-AR with its partly converse effects. Electrophysiological characteristics of TKO mice have not been investigated systematically before.

**Figure 2:**
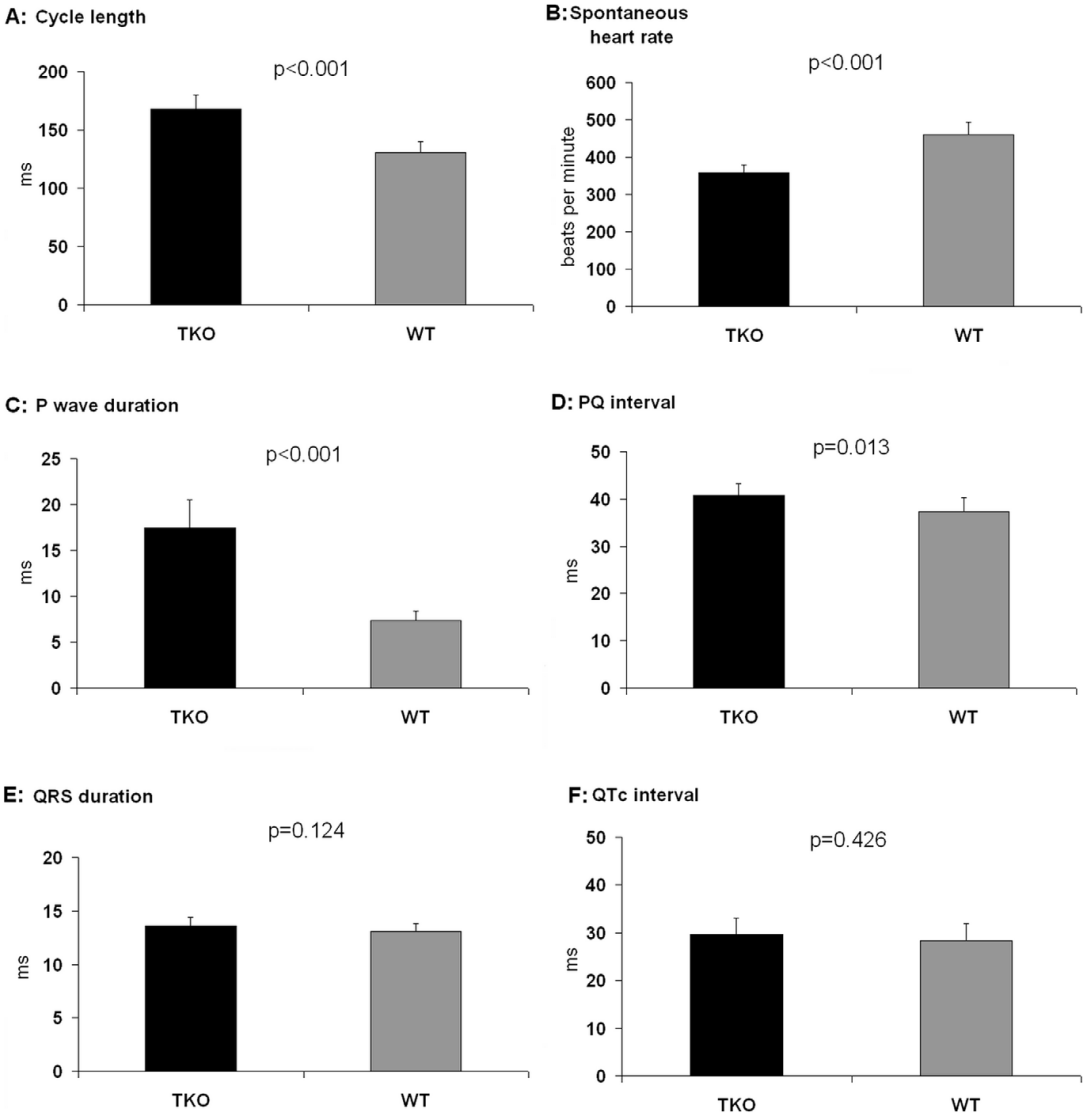
Baseline ECG parameters. Cycle lenghth, spontaneous heart rate, P wave duration and PQ time (A–D) showed a siginificant prolongation in TKO. QRS duration and QTc interval did not differ (E–F). n = 10 for TKO and n = 10 for WT.

## Materials and Methods

### Transgenic animals and Ethics Statement

We performed the studies on 15-week-old mice with a ubiquitous β_1/2/3_-AR knockout. Isogenic SV129 wild type (WT) littermates (β_1_^+/+^β_2_^+/+^β_3_^+/+^) were used as controls. Generation of the TKO-mice has been described previously [17]. The handling of all animals was carried out according to the animal protection law stated in the German civil code and the investigations were approved by the National Office for Nature, Environment and Consumer Protection in Recklinghausen, Nordrhein-Westfalen (Permit Number: 50.203.2-BN 22,22-4 and 8.87-50.10.37.09.272). The mice had free access to water and standard laboratory chow diet and were kept at an artificial light/dark cycle at 20–22°C. The investigation was conform to the Guide for the Care and Use of Laboratory Animals published by the US National Institutes of Health (NIH Publication No. 85–23, revised 1985). No animal died spontaneously before the performance of the electrophysiological investigation.

**Figure 3:**
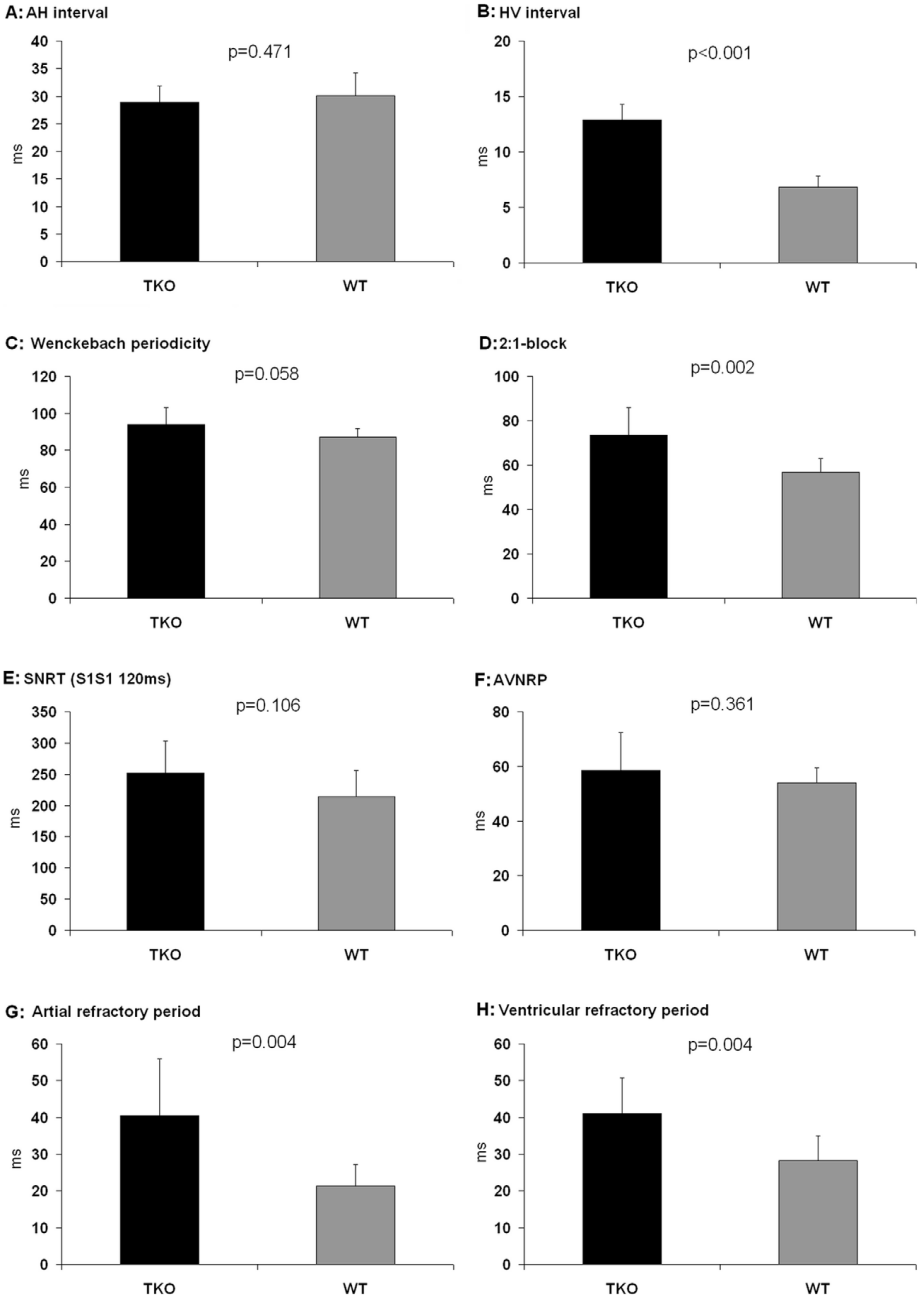
Intracardiac ECG parameters and functional testing during the electrophysiological investigation. Significant differences were found in the infra-Hisian conduction that was markedly delayed in TKO (B). The 2∶1-AV-block occurred at significantly slower fixed atrial pacing cycle lengths in TKO (D). Both the atrial and the ventricular refractory period demonstrated significant prolongation in TKO (G–H). AH: Interval from first atrial signal to His signal. HV: Interval from His to first intracardiac ventricular signal. 2∶1-block: 2∶1-AV-nodal-block. SNRT: sinus node recovery time. AVNRP: AV nodal refractory period. n = 10 for TKO and n = 10 for WT.

### Electrophysiological investigation and surface-ECG

The *in vivo* transvenous electrophysiological investigations were performed in all mice (10 TKO and 10 WT), using a single catheter technique as described before [19], [20]. After initiation of an inhalation anaesthesia with isoflurane the jugular vein was dissected and a 2-French octapolar mouse electrophysiological catheter [eight 0.5 mm circular electrodes; electrode-pair spacing 0.5 mm (Ciber Mouse, NuMed Inc., NY, USA)] was positioned in the right cardiac cavities on atrial and ventricular level. The surface 6-lead ECG was monitored continuously and analyzed under stable conditions for three minutes, as described before [21], [22]. For the whole observation time the incidence of supraventricular and ventricular ectopic beats was evaluated qualitatively. All data were amplified, filtered, sampled at 4 kHz and digitally stored (LabSystem,C.R. Bard Inc., New Jersey, USA). The rate corrected QT-interval (QTc) was calculated according to Mitchell et al [23].

**Figure 4:**
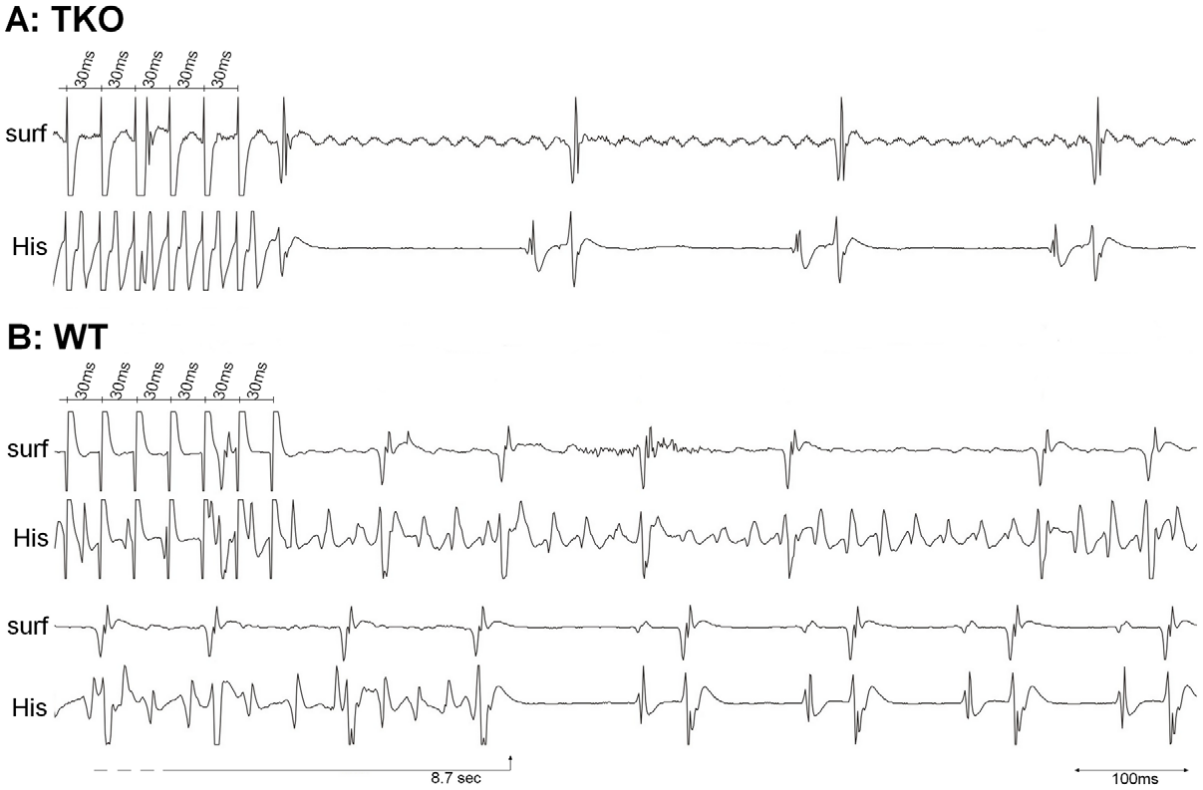
Induction of atrial fibrillation. Representative tracings of A: TKO mice and B: WT mice. Atrial burst stimulation close to the refractory period (S1S1: 30 ms) failed to induce atrial fibrillation (AF) in TKO. The same burst led to the induction of an AF episode in WT that terminated spontaneously after 8.7 seconds. Surf: Surface ECG. His: Intracardiac ECG close to His bundle.

The registration and recording of the intracardiac electrograms and transvenous atrial and ventricular stimulation maneuvers were carried out as previously described [19], [20]. We analysed the intracardiac ECG for AH (interval from first atrial signal to His signal) and HV (interval from His to first ventricular signal) time as surrogates for supra- and infra-Hisian conductivity, respectively.

**Figure 5:**
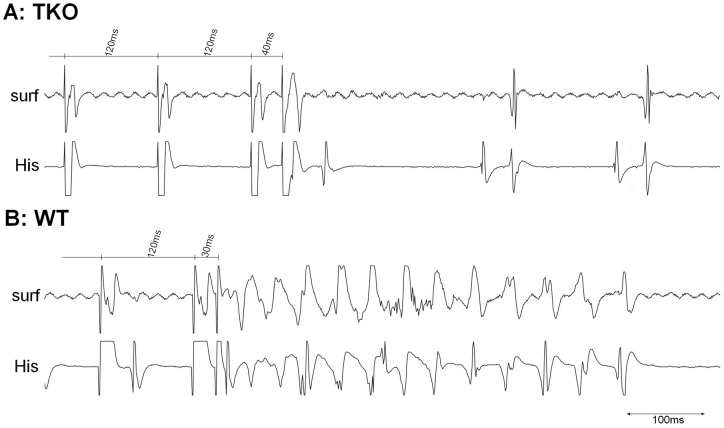
Induction of ventricular tachycardias. Representative tracings of A: TKO mice and B: WT mice. Ventricular extrastimulus (VES) pacing close to the ventricular refractory time did not lead to ventricular tachycardias (VTs) in TKO mice (S1S2: 40 ms). In WT a short VT was induced by VES pacing (S1S2: 30 ms). Surf: Surface ECG. His: Intracardiac ECG close to HIS bundle.

**Figure 6:**
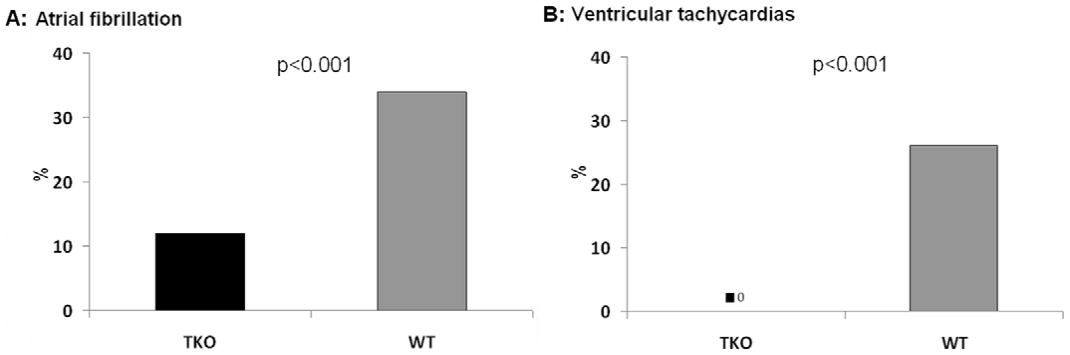
Probability of induction of arrhythmias. The probability of induction of AF was significantly lower in TKO compared to WT. The probability of induction of VTs was highly significant lower in TKO as in this group not any VTs could be induced during the electrophysiological study. n = 10 for TKO and n = 10 for WT.

Functional electrophysiological parameters were determined using a modified multi-programmable simulator (Model 5328; Medtronic, MN, USA). Sinus node recovery time (SNRT) was calculated after fixed-rate atrial pacing and defined as the maximum return cycle length after 10 s fixed-rate pacing at S1S1 cycle length 120 ms. Wenckebach periodicity (WBP) and 2∶1-AV-nodal block (2∶1-block) were evaluated by fixed rate atrial pacing (10 s fixed rate at S1S1: 120 ms, 5 ms stepwise reduction). WBP was defined as longest S1S1 cycle length with loss of 1∶1-AV-nodal conduction; 2∶1-block as longest S1S1 with 2∶1 AV-nodal conduction.

Performing programmed stimulation maneuvers (7 stimuli fixed rate at S1S1 cycle length: 120 ms; one short coupled extrastimulus with a 5 ms stepwise S1S2 reduction), atrial, ventricular and AV nodal refractory periods (ARP, VRP and AVNRP) were evaluated. ARP was defined as longest S1S2 with absent atrial response, AVNRP as longest S1S2 with loss of AV-nodal conduction, and determined after programmed atrial stimulation. VRP was measured analogously after ventricular extrastimulus pacing.

### Arrhythmia induction

The induction of atrial fibrillation (AF) was tested by atrial burst stimulation (5s at S1S1: 50–10 ms, 10 ms stepwise reduction; stimulus amplitudes 1.0 and 2.0 mA). Rapid and fragmented atrial electrograms with irregular AV-nodal conduction for ≥1 s were defined as AF [20], [24]. Ventricular burst stimulation (1 s at S1S1: 50–10 ms, 10 ms stepwise reduction; stimulus amplitudes 1.0 and 2.0 mA) and ventricular extrastimulus pacing (S1S1: 120 ms, 100 ms, and 80 ms followed by up to 3 extra beats) were performed to evaluate ventricular vulnerability. Ventricular tachycardia (VT) was defined as ≥4 ventricular ectopic beats. The probabilities of induction of AF (10 testings per animal) and VT (19 testings per animal) were analyzed and defined as number of inducible arrhythmia episodes divided by the number of total testing maneuvers applied to all animals [20].

### Statistical analysis

Data were expressed as mean ± one standard deviation. Differences between the two groups were assessed using a two-tailed Student's t-test. Discrete variables were analyzed by 2-sided Fisher's exact test. A Spearman nonparametric test was performed to assess the correlation of refractory periods and episodes of arrythmias. A p-value <0.05 was regarded as statistically significant.

## Results

### Surface-ECG

During the whole observation time of the surface ECG all animals presented with sinus rhythm without spontaneous supraventricular or ventricular premature contractions. No atrio-ventricular conduction blocks could be registered. Representative ECG recordings are presented in Figure 1.

In TKO mice we found a significant reduction in heart rate (359.2±20.9 bpm vs. 461.1±33.3 bpm; p<0.001) and significantly prolonged parameters for P wave duration (17.5±3.0 ms vs. 15.1±1.2 ms; p = 0.019), PQ interval (40.8±2.4 ms vs. 37.3±3.0 ms; p = 0.013) and QT time (38.3±4.6 ms vs. 33.5±1.6 ms; p = 0.003) compared to WT (Figure 2). QRS interval (13.6±0.8 ms vs. 13.1±0.7 ms; p = 0.124) as well as the rate corrected QTc time (29.6±3.4 ms vs. 28.3±3.6 ms; p = 0.426) revealed no differences between the groups.

### Electrophysiological investigation

Intracardiac ECG recordings showed equal supra-Hisian (AH, 28.9±2.9 ms vs. 30.1±4.1 ms; p = 0.471) but prolonged infra-Hisian (HV, 12.9±1.4 ms vs. 6.8±1.0 ms; p<0.001) conduction times in TKO compared to WT (Figure 3A and 3B). Functional testing did not demonstrate any differences in SNRT (251.4±51.5 ms vs. 214.4 ± 41.5 ms; p = 0.106) and AVNRP (58.5 ± 13.8 ms vs. 53.9±5.5 ms; p = 0.361). The moderate stress as a result of a faster pacing rate for testing WBP substantiated a trend towards impaired conductance in TKO (94.0±9.0 ms vs. 87.2±4.4 ms; p = 0.058); the 2∶1-block appeared at significantly slower fixed atrial pacing cycle lengths in TKO (73.5±12.5 ms vs. 56.7±6.1 ms; p = 0.002) as a sign of impaired AV-nodal conductivity (Figure 3C–F). Both the atrial and the ventricular refractory period were significantly prolonged in TKO compared to WT (40.5±15.5 ms vs. 21.3±5.8 ms; p = 0.004; and 41.0±9.7 ms vs. 28.3±6.6; p = 0.004, respectively; Figure 3G-H).

### Arrhythmia induction

The probability of induction of AF (Figure 4) was significantly lower in TKO compared to WT (12% vs. 34%; p<0.001). All AF episodes in TKO were short (<1 min) and low in incidence (1.2 episodes per animal). In WT long lasting episodes of AF (>1 minute) could be induced and the overall inducibility of AF episodes was significantly higher (3.4 episodes per animal; p = 0.002).

VTs were inducible in 77.8% of the WT animals with a frequency of 5.0 episodes per animal (Figure 5). In comparison to WT, in TKO there were no VTs inducible at all (p<0.001) (Figure 6). In the inducible WT animals, a significant negative correlation could be found between the VRP and VT-episodes per animal (r = −0.774, p<0.001) that indicated a reduced susceptibility to arrhythmias with longer refractory periods.

## Discussion

β-AR stimulation is a major neurohumoral mechanism in the regulation of the cardiac function [5]. Total loss of β_1/2/3_-AR is conformable with a viable mouse model. This unique model allows the evaluation of a complete β-AR knockout mimicking a total β-AR blockade. To our knowledge there is no data available of cardiac electrophysiology in a setting of complete β-AR blockade. Since human medical therapy with β-blocking agents does not lead to a complete blockade, this mouse model provides an excellent opportunity to further analyse cardiac β-AR function.

The multifarious modes of action of the beta receptors are not yet fully understood. Studies with β_1_-, β_2_-, β_1/2_- and β_3_-AR knockout mice have shown a certain cardiotoxic effect for the β_1_-AR and a cardioprotective effect for the β_2_- and β_3_-AR. Stimulation of the β_1_-AR mediates apoptosis [25] and leads to a diminished contractility and raised arrhythmogenesis by activating proteinkinase A and calcium/calmodulin kinase II, thus leading to increased diastolic calcium leak from the sarcoplasmatic reticulum [26]. An overexpression of the β_1_-AR leads to a more severe cardiomyopathy than overexpression of β_2_-AR [27].

An anti-apoptotic effect is suggested for the β_2_-AR by activation of Gi, PI3K and protein kinase B [28], [29]. Likewise a deletion of the β_2_-AR results in an elevated rate of isoproterenol-induced apoptosis [30]. An in vivo mouse model to evaluate cardiotoxicity by administration of doxorubicin showed that a β_2_-AR knockout but not a β_1_-AR knockout resulted in a rapid death rate due to elevated levels of MAPK [31]. This finding however was dose dependent: using lower doses of doxorubicin a converse result was described [4], demonstrating once again the complexity of β-AR function.

The recently characterised β_3_-AR is also associated with cardioprotective effects by downstream signalling through NO due to activation of eNOS, iNOS and nNOS [9], [32], [33]. The β_3_-ARs are activated only with higher concentrations of catecholamines than β_1_- and β_2_-ARs attributing them a protective function during sympathetic overstimulation [3]. Studies in β_3_-AR knockout mice with pressure-overload by aortic constriction resulted in augmented myocardial fibrosis, hypertrophy, ventricular dilatation and mortality [33]. However, the antagonism of the β_3_-AR did also lead to short term improvement in cardiac function in the setting of animal models with heart failure [34], [35].

Addressing specific parameters of cardiac electrophysiology it could be demonstrated that the single loss of β_1_-AR or the combined loss of β_1_+β_2_-AR led to a decrease in resting heart rate. Moreover the parameter of heart rate variability was predominantly dependent on the β_1_-AR [36]. Single deficiency of β_2_-AR did not lead to differences in resting heart rate [37], [38] or heart rate variability [36]. However, previous studies did not show differences in heart rate of β_1_-AR and β_1_+β_2_-AR knockout mice [39], [40] which was retrospectively attributed to short ECG registration times. Consistent with Ecker et al. [36] we found that a combined β_1/2/3_-deficiency resulted in a markedly reduced heart rate. Moreover we registered prolonged global conduction times on atrial and ventricular levels as well as in the specific conduction system.

### Negative chronotropy and dromotropy

Although TKO mice displayed a reduced ejection fraction in a recent study, no functional impairment in a voluntary running wheel test but rather an increased willingness to run was found [18]. These results were partly explained by a decrease in SERCA 2a-activity and by increased myofibrillar calcium sensitivity in TKO. We further presume that the preserved fitness of TKO results from the reduced heart rate since inter alia heart rate has a major influence on cardiac oxygen consumption [41]. Analogous results in humans have been reported by Eynon et al. who demonstrated that beta-blocker treatment showed no alterations in maximal workload at the anaerobic threshold in a cycle ergometer test despite featuring a reduced heart rate and diminished cardiac output at rest compared to individuals without beta-bockade [42].

In patients with heart disease, Hisian and infra-Hisian conduction delay are associated with increased mortality [43], [44], [45], [46]. We found a prolonged infra-Hisian (HV interval) conductance time in TKO mice. This is consistent with the higher density of β_2_-AR in the cardiomyocytes of the interventricular septum close to the specific cardiac conduction system compared to normal working myocardium [47]. Based on our results we assume that the reported increased mortality is rather a consequence of the concomitant heart disease than the conduction delay in itself as β-AR blockade basically shows positive effects on mortality in patients after myocardial infarction or in patients with congestive heart failure [48], [49].

### Suppression of arrhythmias

Accompanying the known negative inotropy and the now demonstrated conduction delays we found a profound suppression of atrial and ventricular arrhythmias. No signs of heart failure, occurrence of spontaneous AV-blocks, or sudden cardiac deaths were present in this mouse model.

The QT interval as well as the atrial and ventricular refractory period plays an important role in arryhthmogenesis [50]. Chronic medical beta blockade in humans can lead to a prolongation of the QT interval [51]. However, depending on the beta-blocker used, no change [52] or even a shortening of QT interval [53] has also been reported. Zhan et al. [54] pointed out that bisoprolol (a selective β_1_-blocker) and not metoprolol (a β_1/2_-blocker) or carvedilol (which blocks β_1_-, β_2_- and α_1_-receptors) had a positive effect on mortality as a result of a reduction in ventricular arrhythmias in a mouse model of dilated cardiomyopathy. They concluded, that the effect might have been due to a significant shortening of the QT time. Here we could verify that TKO leads to a drastic reduction of the inducibility of VTs even though no changes in QTc interval were present at all. Hence, other mechanisms seem to be responsible for the protective effect found in TKO.

The assumption that the suppression of inducible arrhythmias in TKO is due to a prolonged ARP and VRP is supported by the fact that in chronic medical beta-blocker treatment in humans, atrial and ventricular refractory periods show significant prolongations [55], [56]. Therefore, this is the likely explanation for the protective effect demonstrated in the TKO mouse model.

Regarding antiarrhythmic treatment, a non-selective medical beta blockade might not be inferior to a selective β_1_-AR blockade. Though the question, which beta-blockade is most relevant cannot finally be answered and is certainly highly dependent on the underlying cardiac disease. Undoubtedly the blocking of β_1_-AR in cases of heart failure is cardioprotective and improves cardiovascular outcome [16]. Selective β_2_-AR (but not β_1_) blockade has been found to be highly antiarrhythmic in heart failure due to a reduction of sarcoplasmatic reticulum calcium load [57] and the beta blocker Metoprolol that has recently shown a higher affinity to β_2_-AR than to β_1_-AR [58] has been effectively administered to treat arrhythmias. Differing data exist for the β_3_–AR: in sepsis [59] and heart failure [60] an overexpression of the β_3_-AR can be determined, but whether this increase is a protective response or might even contribute to further myocardial dysfunction is still a matter of debate. The ‘physiological break-mechanism’ of the β_3_-AR to sympathetic overstimulation plays a secondary role in the TKO model as no stimuli of the β_1_- and β_2_-AR can emerge.

Based on our present results, a general recommendation cannot be given to use an unselective complete medical beta blockade to treat arrythmias; however, the data show that TKO mice exhibit a highly antiarrythmogenic potential. Further studies should address the electrophysiological phenotypology of mice with a single β-AR knockout, as well as combined β_1/2_-, β_1/3_-, and β_2/3_-AR knockout to further clarify the role of the antiarrythmic effect of the individual receptor blockade. Moreover, our data provide the basis to further test possible beta-blocker side-effects that might occur independently of the β-AR in a unique mouse model.

## Conclusions

TKO results in significant prolongations of cardiac conduction times and refractory periods. This was accompanied by a highly significant reduction of atrial and ventricular arrhythmias. Our findings confirm the importance of β-AR in arrhythmogenesis and their role as therapeutic target.
